# A unified framework for multi-locus association analysis of both common and rare variants

**DOI:** 10.1186/1471-2164-12-89

**Published:** 2011-01-31

**Authors:** Daniel Shriner, Laura Kelly Vaughan

**Affiliations:** 1 Center for Research on Genomics and Global Health, National Human Genome Research Institute, Bethesda, MD 20892 USA; 2 Department of Biostatistics, Section on Statistical Genetics, University of Alabama at Birmingham, Birmingham, AL 35294 USA

## Abstract

**Background:**

Common, complex diseases are hypothesized to result from a combination of common and rare genetic variants. We developed a unified framework for the joint association testing of both types of variants. Within the framework, we developed a union-intersection test suitable for genome-wide analysis of single nucleotide polymorphisms (SNPs), candidate gene data, as well as medical sequencing data. The union-intersection test is a composite test of association of genotype frequencies and differential correlation among markers.

**Results:**

We demonstrated by computer simulation that the false positive error rate was controlled at the expected level. We also demonstrated scenarios in which the multi-locus test was more powerful than traditional single marker analysis. To illustrate use of the union-intersection test with real data, we analyzed a publically available data set of 319,813 autosomal SNPs genotyped for 938 cases of Parkinson disease and 863 neurologically normal controls for which no genome-wide significant results were found by traditional single marker analysis. We also analyzed an independent follow-up sample of 183 cases and 248 controls for replication.

**Conclusions:**

We identified a single risk haplotype with a directionally consistent effect in both samples in the gene *GAK*, which is involved in clathrin-mediated membrane trafficking. We also found suggestive evidence that directionally inconsistent marginal effects from single marker analysis appeared to result from risk being driven by different haplotypes in the two samples for the genes *SYN3 *and *NGLY1*, which are involved in neurotransmitter release and proteasomal degradation, respectively. These results illustrate the utility of our unified framework for genome-wide association analysis of common, complex diseases.

## Background

Mapping disease susceptibility loci for complex diseases has proven to be challenging. The etiology of a complex disease is multifactorial, with both genetic and environmental risk factors and interactions among these risk factors. Genome-wide association (GWA) studies are an alternative to traditional family-based linkage studies for mapping disease susceptibility loci for complex diseases. The most common design to date for GWA studies is to genotype a dense panel (> 100,000) of single-nucleotide polymorphisms (SNPs) for a large collection of unrelated cases and controls. SNPs are then tested one marker at a time for association most often using contingency table analysis, *i.e*., a *X*^2^test or Fisher's exact test with one or two degrees of freedom, depending on the hypothesis being tested. The resulting set of *p*-values is then adjusted for multiple comparisons, usually a Bonferroni correction for the number of tests performed.

Single marker analysis involves the marginalization of effects over all genetic and environmental backgrounds. Consequently, effect sizes for single markers tend to be small (odds ratios of 1.2 to 1.5 per copy of the risk allele or smaller), necessitating sample sizes of thousands to obtain reasonable levels of power at genome-wide significance levels [1]. Multi-locus methods may have increased power if they account for the correlation of loci due to linkage disequilibrium. However, multi-locus methods generally have been observed to have decreased power because they tend to produce test statistics with larger degrees of freedom [2]. Therefore, in designing a powerful and efficient multi-locus method for generating testable hypotheses based on genome-wide SNP data, we are motivated to reduce the degrees of freedom.

Alternatively, consider a biochemical pathway consisting of many genes, which may be located on different chromosomes and hence may segregate independently. This motivation raises the general question of how to test a set of markers that may be biologically related but not correlated via linkage disequilibrium. Possibilities include multivariate techniques such as the F test or multiple regression. However, these techniques can also lose power due to large degrees of freedom.

A third issue for association analysis is low power for rare variants that medical sequencing is expected to uncover. To overcome low power for variants with frequencies < 5%, specialized methods for grouping variants have been described [3,4], but such methods were designed only for analysis of rare variants for which linkage disequilibrium is expected to be negligible. Furthermore, the two most commonly used estimators of linkage disequilibrium, *r*^2 ^and *D*', are biased upwards when allele frequencies are low or sample sizes are small [5,6].

In response to all of these motivations, we propose a flexible multi-locus method based on unions of multiple SNPs that yields a test that always has only one degree of freedom. The method implicitly accounts for linkage disequilibrium and is appropriate for simultaneous analysis of common and rare variants. We demonstrate by simulation that our method has valid control of the false positive error rate while yielding more power than traditional single marker analysis. We also demonstrate by simulation that our method is sensitive to any source of differential correlation among markers. We then analyze two publicly available data sets for Parkinson disease (PD) for which no significant associations were detected by single marker analysis [7-9]. Using our new method, we found evidence supporting susceptibility to PD at three loci.

## Methods

### Study Samples

Parkinson disease (MIM 168600, http://www.ncbi.nlm.nih.gov/omim) is the second most common neurodegenerative disorder after Alzheimer disease. For the discovery sample, we obtained publicly available data from the PROGENI and GenePD studies of familial PD [9]. Briefly, 2,082 cases and controls were genotyped using Illumina HumanCNV370 version1_C BeadChips (Illumina, San Diego, CA) and the Illumina Infinium II assay protocol.

For the replication sample, we obtained publicly available data from a study of sporadic PD from the SNP Resource at the National Institute of Neurological Disorders and Stroke Human Genetics Resource Center DNA and Cell Line Repository http://ccr.coriell.org/ninds/ as well as clinical data [7,8]. The original genotyping was performed in the laboratory of Drs. Andrew Singleton and John A. Hardy (National Institute on Aging, Laboratory of Neurogenetics), Bethesda, MD USA. Briefly, 408,803 SNPs were genotyped using the Illumina Infinium I and HumanHap300 assays on 270 cases and 270 neurologically normal controls. The data made available had already been processed for quality control [7].

### The Framework of Logical Unions

To illustrate the construction of a union based on classical (binary) logic, consider two events. Let *P*(*X*) be the probability of event *X *and *P*(*Y*) be the probability of event *Y*. The probability of the union of events *X *and *Y*, *p *(*X *∪ *Y*), is *P *(*X *∪ *Y*) = *P *(*X*) + *P *(*Y*) - *P *(*X *∩ *Y*). To estimate the probability of the intersection *P *(*X *∩ *Y*), we need a measure of correlation. One commonly used measure of correlation is the pairwise correlation coefficient, given by $r=\frac{\text{cov}\left(X,Y\right)}{\sqrt{\text{var}\left(X\right)}\sqrt{\text{var}\left(Y\right)}}=\frac{P\left(X\cap Y\right)-P\left(X\right)P\left(Y\right)}{\sqrt{P\left(X\right)\left(1-P\left(X\right)\right)}\sqrt{P\left(Y\right)\left(1-P\left(Y\right)\right)}}$. After rearrangement and substitution, $P\left(X\cup Y\right)=P\left(X\right)+P\left(Y\right)-P\left(X\right)P\left(Y\right)-r\sqrt{P\left(X\right)\left(1-P\left(X\right)\right)P\left(Y\right)\left(1-P\left(Y\right)\right)}$.

In the genetic context, let *A *represent the major allele and *B *represent the minor allele at a SNP. With respect to the minor allele, dominant coding of the three genotypes (*AA,AB,BB*) is given by (0,1,1) and recessive coding is given by (0,0,1). Additive coding of the number of copies of the minor allele, *i.e*.,(0,1,2), can be achieved by summing over dominant and recessive variables since (0,1,1) + (0,0,1) = (0,1,2) and thus need not be separately considered [10]. *r*^2 ^is a commonly used measure of linkage disequilibrium. Thus, unions implicitly account for correlation between markers, including coupling (*r *> 0) or repulsion (*r *< 0) phase. It is critical to note that this use of the correlation coefficient encompasses gametic phase disequilibrium as well as other sources of correlation such as epistasis and natural selection. Also note that, by definition, *P *(*X *∪ *X*) = *P*(*X*), demonstrating the equivalence of analysis of a union of one marker with traditional single marker analysis.

There are many possible coding schemes one could implement within this framework. In this study, we construct unions over consecutive, non-overlapping sets of *k *markers. The choice of non-overlapping sets was made to facilitate calculating the genome-wide testing burden. Let *x_ij _*be a binary indicator variable for the *i*^th ^individual and the *j*^th ^union. With two markers (loci denoted by subscripts) under dominant coding for the minor allele,

$$x_{ij}=\{\begin{array}{lll} 0 & \text{for genotype} & A_{1}A_{1}A_{2}A_{2}\\ 1 & \text{for genotypes} & A_{1}B_{1}A_{2}A_{2},B_{1}B_{1}A_{2}A_{2},A_{1}A_{1}A_{2}B_{2},A_{1}A_{1}B_{2}B_{2},A_{1}B_{1}A_{2}B_{2},A_{1}B_{1}B_{2}B_{2},B_{1}B_{1}A_{2}B_{2},B_{1}B_{1}B_{2}B_{2}\\ 1 & \text{for genotypes} & A_{1}B_{1}{\text{?}}_{2}{\text{?}}_{2},B_{1}B_{1}{\text{?}}_{2}{\text{?}}_{2},{\text{?}}_{1}{\text{?}}_{1}A_{2}B_{2},{\text{?}}_{1}{\text{?}}_{1}B_{2}B_{2}\\ \text{?} & \text{for genotypes} & A_{1}A_{1}{\text{?}}_{2}{\text{?}}_{2},{\text{?}}_{1}{\text{?}}_{1}A_{2}A_{2},{\text{?}}_{1}{\text{?}}_{1}{\text{?}}_{2}{\text{?}}_{2} \end{array},$$

in which ? represents missing data. Under dominant coding, a value of 0 indicates the absence of at least one *B *allele (the null hypothesis is the intersection) and a value of 1 indicates the presence of at least one *B *allele (the alternative hypothesis is the union). Similarly, with two markers under recessive coding for the minor allele,

$$x_{ij}=\{\begin{array}{lll} 0 & \text{for genotypes} & A_{1}A_{1}A_{2}A_{2},A_{1}A_{1}A_{2}B_{2},A_{1}B_{1}A_{2}A_{2},A_{1}B_{1}A_{2}B_{2}\\ 1 & \text{for genotypes} & A_{1}A_{1}B_{2}B_{2},A_{1}B_{1}B_{2}B_{2},B_{1}B_{1}A_{2}A_{2},B_{1}B_{1}A_{2}B_{2},B_{1}B_{1}B_{2}B_{2}\\ 1 & \text{for genotypes} & B_{1}B_{1}{\text{?}}_{2}{\text{?}}_{2},{\text{?}}_{1}{\text{?}}_{1}B_{2}B_{2}\\ \text{?} & \text{for genotypes} & A_{1}A_{1}{\text{?}}_{2}{\text{?}}_{2},A_{1}B_{1}{\text{?}}_{2}{\text{?}}_{2},{\text{?}}_{1}{\text{?}}_{1}A_{2}A_{2},{\text{?}}_{1}{\text{?}}_{1}A_{2}B_{2},{\text{?}}_{1}{\text{?}}_{1}{\text{?}}_{2}{\text{?}}_{2} \end{array}$$

Under recessive coding, a value of 0 indicates the absence of at least one *BB *genotype (the null hypothesis is the intersection) and a value of 1 indicates the presence of at least one *BB *genotype (the alternative hypothesis is the union). Any individual for which *x_ij _*= ? is removed from analysis of the *j*^th ^union. Note that this choice of implementation relies solely on counting individuals with pre-specified multi-locus genotypes, does not require estimation of *p_i_*, *p_j_*, or *r*, and does not assume Hardy-Weinberg equilibrium.

For case-control data, we test for association using Fisher's exact test. For the *j*^th ^union, the 2 × 2 contingency table is constructed by counting the numbers of individuals for which *x_ij _*is 0 or 1 among cases and controls. The test has only one degree of freedom, regardless of *k*. We control for multiple comparisons using a Bonferroni correction for the number of unions tested. The number of unions tested is given by $\left\lceil \frac{\#SNPs}{k}\right\rceil$, in which ┌x┐ is the ceiling function and returns the smallest integer not less than *x*. This implementation of multiplicity control preserves more power as *k *increases and as the testing burden is consequently reduced. Given 319,813 SNPs in the discovery sample and accounting for both dominant and recessive coding over unions of size one to five, the genome-wide significance level was $\frac{0.05}{2\times \sum_{k=1}^{5}\left\lceil \frac{319,813}{k}\right\rceil }=3.42\times 10^{-8}$. All union testing was performed in R [11].

### Other Analyses

Imputation was performed using MACH, version 1.0.16 http://www.sph.umich.edu/csg/abecasis/MACH/download/. For the reference panel, we retrieved the combined HapMap phase II+III raw genotype files for the CEU sample http://hapmap.ncbi.nlm.nih.gov/downloads/genotypes/latest_phaseII+III_ncbi_b36/forward/non-redundant/. We filtered the 3,907,239 autosomal CEU SNPs based on the inclusion of unrelated individuals only, a minor allele frequency ≥ 0.01, a SNP missingness rate ≤ 5%, and an individual missingness rate ≤ 5%. We inferred haplotype phases for the reference data using the settings --rounds 50 --states 200. We conditioned imputation on the maximum-likelihood estimates of the crossover map and the error rate map. We retained all imputed genotype calls for which the posterior probability ≥ 0.9.

We performed haplotype analysis using PLINK version 1.06 http://pngu.mgh.harvard.edu/purcell/plink/[12]. Briefly, we compared each haplotype against all of the other haplotypes.

## Results

### Simulation Analysis

**Figure 1:**
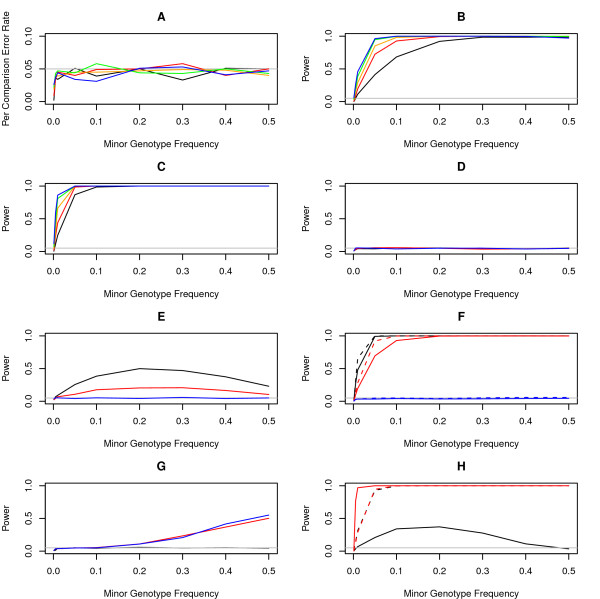
**Validity and power analysis of union testing**. Each simulated data set consisted of 938 cases and 863 controls. For each experiment, 1,000 independent replicates were simulated. Coded genotypes were simulated by randomly sampling from a binomial distribution with the given frequencies. Case-control status was determined by a logistic model. Gray lines indicate the per comparison significance threshold of 0.05. A) Per comparison error rate. Unions of one, two, three, four, and five SNPs are represented by black, red, orange, green, and blue lines, respectively. B) Power for an odds ratio of 1.5 for each SNP. C) Power for an odds ratio of 2 for each SNP. D) Power for 2-marker unions with opposing effects. The black line represents odds ratios of 2 and 0.5 for the two markers, the red line represents 1.5 and 0.67, and the blue line represents 1 and 1. E) Power for unions consisting of one predictor with an odds ratio of 2 (black line), 1.5 (red line), or 1 (blue line), and four predictors with odds ratios of 1. F) Power for 2-marker unions with correlated predictors. Solid lines represent independent predictors and dotted lines represent predictors correlated at *r*^2 ^= 0.8. Black lines represent odds ratios of 2, red lines represent 1.5, and blue lines represent 1. G) Power to detect epistasis for 2-marker unions. The black line represents odds ratios of 1 for both markers and 1 for the epistatic effect. The red line represents odds ratios of 1 for both markers and 2 for the epistatic effect. The blue line represents odds ratios of 1 for both markers and 0.5 for the epistatic effect. H) Power to detect differential correlation between 2-marker unions. Black lines represent *r*^2 ^= 0 in controls and *r*^2 ^= 0.8 in cases. Red lines represent *r*^2 ^= 0.8 in controls and *r*^2 ^= 0 in cases. Solid lines represent odds ratios of 2 and dotted lines represent 1.

We first investigated the validity and power of our proposed method, exploring over sizes of unions of SNPs ranging from one to five. Note that analysis of a union of one SNP is identical to single marker analysis. Under the null hypothesis of no association, analysis of unions has the expected per comparison error rate and is therefore valid (Figure 1A). For small effect sizes (odds ratios ≤ 2), analysis of unions is increasingly more powerful than single marker analysis as the frequency of the minor multi-locus genotype decreases (Figs. 1B and 1C). If a union consists of both risk-increasing and risk-decreasing predictors, effects will cancel and power will be lost (Figure 1D). Also, if a union consists of too many predictors with no effect on the outcome, then power to detect a predictor with an effect within the union will decrease (Figure 1E). Analysis of unions of correlated predictors can be more powerful than analysis of unions of independent predictors (Figure 1F). Epistasis (nonadditivity on the logit scale) can either increase or decrease power, depending on the directions of the effect sizes for epistatic effects *vs*. marginal effects (Figure 1G). For epistasis to be detectable, minor genotype frequencies must be large so that the joint genotype counts are reasonably large.

The union test is a valid test of association of genotype frequencies if the correlation between markers is matched in cases *vs*. controls (Figure 1F). The union test is also a powerful test of differential correlation among markers, even in the absence of differences in genotype frequencies (Figure 1H). Thus, the union test can simultaneously detect differences in genotype frequencies, epistasis, haplotype structure, natural selection, population structure, and any other process affecting correlation among markers.

### Comparison to Other Methods

We compared the union test to a logistic kernel machine-based test, a set-based test for common variant analysis that adaptively estimates the degrees of freedom given the correlational structure of the markers in the set [2,13]. Both tests were valid at the 0.05 significance level (Additional File 1). The union test was more powerful than the kernel machine-based test for multiple independent markers with nonzero effects (Additional File 2 and Additional File 3). The union test was also more powerful than the kernel machine-based test when the minor genotype frequency was below ~0.10 (Additional File 2 and Additional File 3). The kernel machine-based test was more powerful than the union test for markers with opposing effects (Additional File 4), dilute signal (Additional File 5), correlated predictors (Additional File 6), and epistasis (Additional File 7). The kernel machine-based test was conservative in the presence of differential correlation between markers under the null hypothesis and had no power to detect differential correlation under the alternative hypothesis (Additional File 8).

We also compared the union test to the collapsing method [3], a set-based test for rare variant analysis that collapses the data into a *X*^2 ^test with one degree of freedom. The collapsing method is based on aggregating genotypes across all loci in the set such that "an individual is coded as 1 if a rare allele is present at any of the variant sites and as 0 otherwise" [3]. Thus, the collapsing method is in fact identical to the union test (under dominant coding) by construction and power is equivalent for both tests. The two main differences between the collapsing method and the union test are that the former assumes that minor allele frequencies are low, *i.e*., ≤ 0.05, and that all markers are independent, *i.e*., there is no linkage disequilibrium (*r*^2 ^= 0) [3]. The collapsing method is thus a special case of the union test, with the union test applicable across the entire range of frequencies and across the entire range of linkage disequilibrium.

### Real Data Analysis

**Figure 2:**
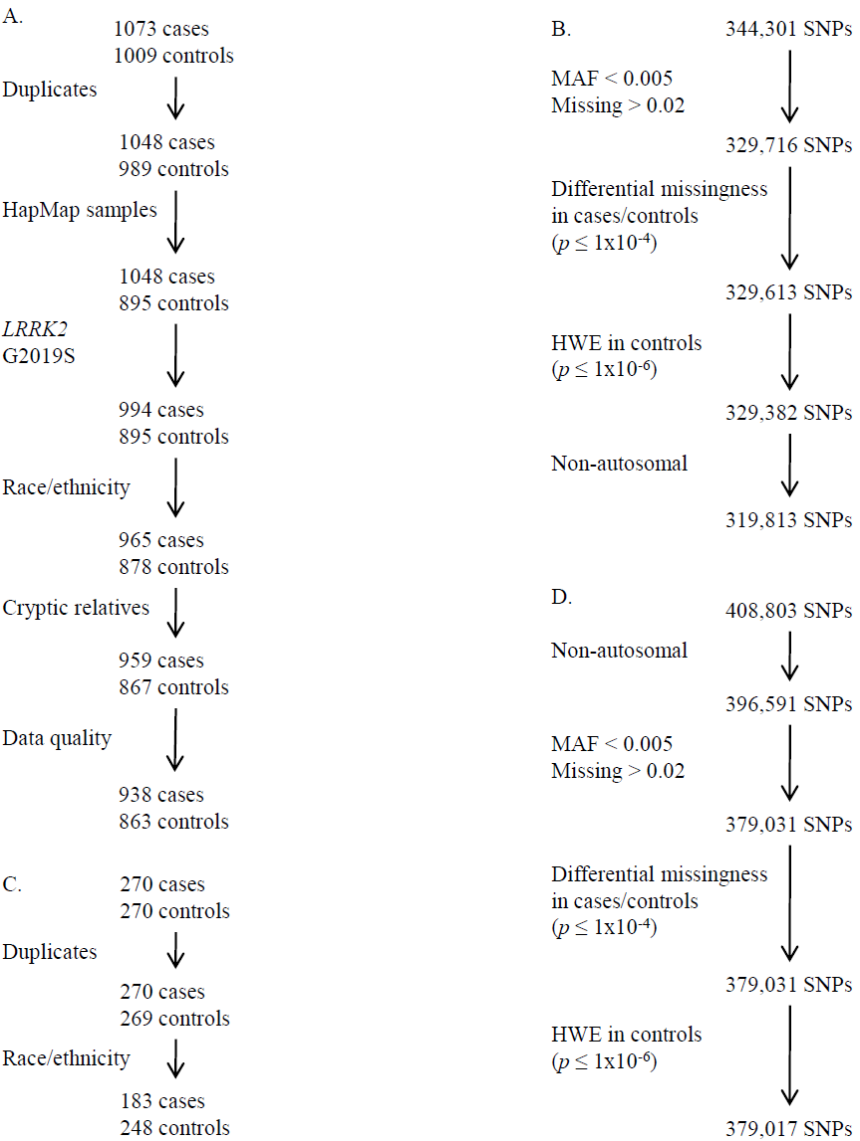
**Quality control**. A) Sample processing for the discovery sample. B) SNP processing for the discovery sample. C) Sample processing for the replication sample. D) SNP processing for the replication sample.

Data processing for quality control for both samples is depicted in Figure 2. For the discovery sample, we retained 938 of the 1,073 cases, 863 of the 1,009 controls, and 319,813 of the 344,301 SNPs. For the replication sample, we retained 183 of the 270 cases, 248 of the 270 controls, and 379,017 of the 408,803 SNPs. To investigate the possibility of population stratification in the discovery and replication samples, we estimated the variance inflation factor of the genomic control method [14,15]. We estimated an inflation factor of 1.05 for the discovery sample and 1.01 for the replication sample, indicating a negligible inflation of the false positive error rate due to population stratification (Additional File 9).

**Figure 3:**
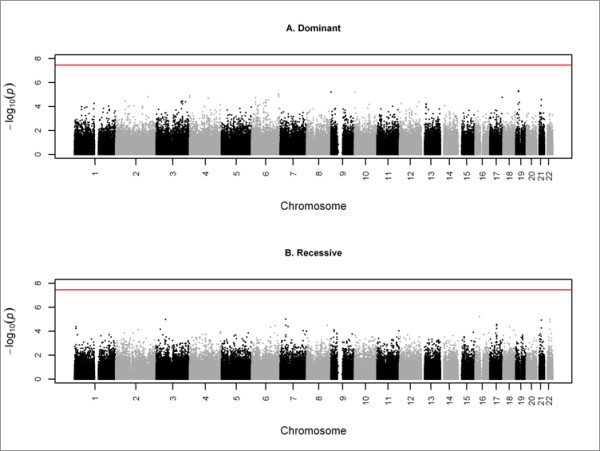
**Genome-wide scans for single marker analysis**. The red horizontal line indicates the significance level of 3.42 × 10^-8^.

Under dominant coding, we identified no regions correlated with PD case/control status at a genome-wide significance level of 3.42 × 10^-8 ^(Figure 3A). We identified seven independent loci at suggestive levels of association (Table 1). Of these, we replicated the region on chromosome 4p16 including the gene *GAK*. This region was discovered using unions of one, two, or three markers but was replicated only in the single marker analysis (Table 1). Using haplotype analysis, we found that the association in both samples was driven by the same low-frequency haplotype (TT) with an odds ratio of 1.58 (Table 2).

**Table 1:** Summary of discovery and replication results

Model	Size	Discovery *P*-value	OR (95% CI)	Replication *P*-value	OR (95% CI)	rsid	Chr	Position (bp)	Minor/major allele	Gene
Dominant	1	8.17 × 10^-6^	0.65 (0.54, 0.79)	0.845	1.04 (0.69, 1.56)	rs1584586	3	151677041	A/G	*TSC22D2*
Dominant	1	6.70 × 10^-6^	1.71 (1.35, 2.18)	0.037	1.69 (1.02, 2.81)	rs1564282	4	842313	T/C	*GAK*
Dominant	1	4.03 × 10^-6^	1.73 (1.36, 2.21)	NA	NA	rs11248051	4	848332	T/C	*GAK*
Dominant	1	8.25 × 10^-6^	1.67 (1.32, 2.11)	0.033	1.66 (1.02, 2.71)	rs11248060	4	954359	T/C	*GAK*
Dominant	1	1.89 × 10^-6^	12.95 (3.24, 112.76)	NA	NA	rs7848576	9	697463	G/A	*ANKRD15*
Dominant	1	3.37 × 10^-6^	0.64 (0.53, 0.78)	0.560	0.89 (0.60, 1.33)	rs898528	17	74678398	T/C	NA
Dominant	1	6.84 × 10^-6^	0.65 (0.54, 0.79)	0.283	1.25 (0.84, 1.87)	rs2830713	21	27416311	T/C	NA
Dominant	2	1.77 × 10^-6^	1.58 (1.30, 1.91)	0.202	1.30 (0.87, 1.95)	rs1564282	4	842313	T/C	*GAK*
						rs2061846	4	842484	C/T	*GAK*
Dominant	2	2.24 × 10^-6^	1.57 (1.30, 1.90)	NA	NA	rs4690339	4	844712	G/A	*GAK*
						rs11248051	4	848332	T/C	*GAK*
Dominant	2	2.91 × 10^-6^	0.63 (0.52, 0.77)	0.252	1.28 (0.84, 1.97)	rs194907	6	82485214	G/A	*FAM46A*
						rs1276888	6	82489107	T/C	*FAM46A*
Dominant	3	1.40 × 10^-6^	1.58 (1.31, 1.92)	NA	NA	rs1564282	4	842313	T/C	*GAK*
						rs2061846	4	842484	C/T	*GAK*
						rs4690339	4	844712	G/A	*GAK*
Dominant	3	9.55 × 10^-6^	0.62 (0.50, 0.77)	1.000	1.01 (0.65, 1.58)	rs1881747	10	54003581	C/T	NA
						rs1919764	10	54015996	C/T	NA
						rs1919738	10	54021111	A/G	NA
Dominant	4	5.06 × 10^-6^	0.61 (0.49, 0.76)	0.914	0.98 (0.63, 1.54)	rs7085224	10	53971746	G/A	NA
						rs1881747	10	54003581	C/T	NA
						rs1919764	10	54015996	C/T	NA
						rs1919738	10	54021111	A/G	NA
Recessive	1	7.25 × 10^-6^	0.22 (0.09, 0.46)	0.060	3.72 (0.88, 22.09)	rs9310784	3	25905208	C/T	*NGLY1*
Recessive	1	2.26 × 10^-6^	1.65 (1.33, 2.04)	0.182	0.73 (0.45, 1.16)	rs2382722	16	27300127	G/A	*IL4R*, *IL21R*
Recessive	1	5.36 × 10^-6^	0.56 (0.43, 0.72)	0.078	1.65 (0.94, 2.91)	rs1159220	22	31410753	T/C	*SYN3*
Recessive	1	6.14 × 10^-6^	0.56 (0.43, 0.73)	0.078	1.65 (0.94, 2.91)	rs3788483	22	31414345	C/T	*SYN3*
Recessive	2	8.33 × 10^-6^	2.06 (1.48, 2.90)	0.521	1.26 (0.64, 2.48)	rs2189387	17	36293632	A/G	*KRT20*
						rs7212483	17	36294578	T/C	*KRT20*
Recessive	2	7.75 × 10^-6^	0.57 (0.44, 0.74)	0.164	1.45 (0.86, 2.45)	rs1159220	22	31410753	T/C	*SYN3*
						rs5998577	22	31412043	A/G	*SYN3*

**Table 2:** Haplotype analysis for the locus at chromosome 4p16

	Discovery			Replication		
**Haplotype ^a^**	**Frequency**	**Odds Ratio**	***P*-value**	**Frequency**	**Odds Ratio**	***P*-value**

CC	0.173	1.17	0.075	0.155	0.92	0.634
TT	0.111	1.59	2.10 × 10^-5^	0.102	1.58	0.049
CT	0.716	0.72	9.86 × 10^-6^	0.743	0.87	0.356

^a ^In order, the SNPs are rs1564282 and rs2061846.

**Figure 4:**
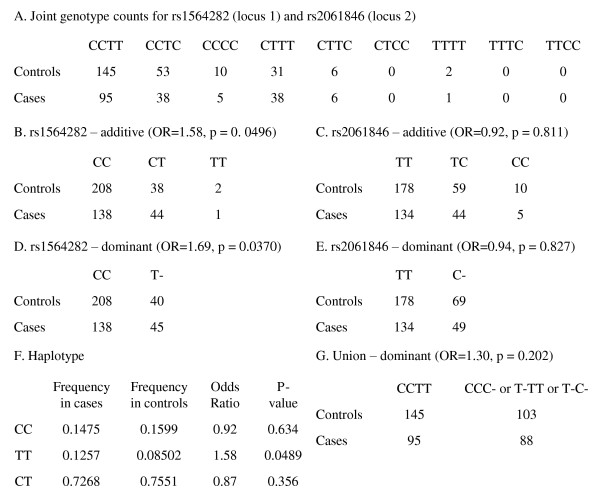
**Comparison of single marker and multi-locus methods**. A) The observed joint genotype counts for rs1564282 and rs2061846. One control with the CT genotype at rs1564282 had a missing genotype at rs2061846. B) Single marker analysis for rs1564282 under additive coding. C) Single marker analysis for rs2061846 under additive coding. D) Single marker analysis of rs1564282 under dominant coding. E) Single marker analysis of rs2061846 under dominant coding. "-" indicates either allele. F) Haplotype analysis. G) Union analysis under dominant coding.

To directly compare single marker analysis, haplotype analysis, and our multi-locus method, we examined the first multi-locus union in Table 1, which consisted of SNPs rs1564282 and rs2061846, using the replication sample (Figure 4A). By 2 × 3 contingency table analysis of genotypes, rs1564282 was significantly associated with PD (Figure 4B) but rs2061846 was not (Figure 4C). Similarly, by 2 × 2 contingency table analysis under dominant coding, rs1564282 was significantly associated with PD (Figure 4D) but rs2061846 was not (Figure 4E). Haplotype analysis revealed significant association with PD for the haplotype consisting of the minor allele at rs1564282 and the major allele at rs2061846, *i.e*., haplotype TT *vs*. haplotypes CC and CT with haplotype TC being unobserved (Figure 4F). Under dominant coding, 2 × 2 contingency table analysis of the union did not yield significant association, because the union tested multi-locus genotype CCTT *vs*. the other eight genotypes, effectively attenuating the signal of haplotype TT (Figure 4G).

In general, for a union over *k *SNPs, the number of joint genotypes is 3*^k ^*and 2 × 2 contingency table analysis is more powerful than 2 × 3*^k ^*contingency table analysis. More importantly, as *k *increases, some joint genotypes likely will be unobserved, leading to cells in the contingency table having counts of zero. Collapsing a 2 × 3*^k ^*contingency table into a 2 × 2 contingency table is one way to address this problem of data sparsity. In our implementation of dominant coding over multiple loci, the reference multi-locus genotype consists solely of diplotypes of the most common haplotype. This example (Figure 4) illustrates the trade-off of gaining power by reducing the degrees of freedom *vs*. losing power by attenuation of signal.

Under recessive coding, we identified no regions correlated with PD case/control status at a genome-wide significance level of 3.42 × 10^-8 ^(Figure 3B). At suggestive significance levels, we identified two potentially interesting loci at chromosomes 3p24 and 22q12 (Table 1). At chromosome 3p24, SNP rs9310784 was associated with decreased risk in the discovery sample but with increased risk in the replication sample. We investigated this directional inconsistency locus using haplotype analysis (Table 3). We identified a haplotype block of eight SNPs including rs9310784. In the discovery sample, the haplotype with the strongest effect carried the minor allele at rs9310784 and was associated with decreased risk. In the replication sample, the haplotype with the strongest effect carried the major allele and was associated with increased risk. Thus, the effects were directionally consistent at the haplotypic level with respect to rs9310784 but were directionally inconsistent with respect to marginal effects at this SNP. At chromosome 22q12, the marginal effects for SNP rs1159220 were directionally inconsistent (Table 1) and different haplotypes drove association in the two samples (Table 4).

**Table 3:** Haplotype analysis for the locus at chromosome 3p24

	Discovery			Replication		
**Haplotype ^a^**	**Frequency**	**Odds Ratio**	***P*-value**	**Frequency**	**Odds Ratio**	***P*-value**

ACGCCAAT	0.799	0.98	0.835	0.782	0.83	0.248
GTATTGCC	0.072	0.76	0.040	0.105	1.01	0.974
GTATTAAT	0.033	1.42	0.069	0.043	0.73	0.365
GCACTACT	NA	NA	NA	0.014	7.11	0.012
GTATTACC	0.033	0.99	0.969	0.041	2.07	0.058
GCATCACC	0.038	1.27	0.195	NA	NA	NA

^a ^In order, the SNPs are rs4481118, rs4293672, rs6551000, rs6793031, rs1991332, rs7644516, rs2052760, and rs9310784.

**Table 4:** Haplotype analysis for the locus at chromosome 22q12

	Discovery			Replication		
**Haplotype ^a^**	**Frequency**	**Odds Ratio**	***P*-value**	**Frequency**	**Odds Ratio**	***P*-value**

TA	0.163	0.84	0.058	0.166	0.99	0.941
CA	0.036	0.93	0.717	0.049	0.49	0.084
TG	0.242	0.89	0.155	0.259	1.21	0.259
CG	0.559	1.20	0.007	0.526	0.97	0.820

^a ^In order, the SNPs are rs1159220 and rs5998577.

Finally, we examined execution time for the union test. Algorithmically, the union test consists of one hypothesis test per union, regardless of the number of markers in the union. For a union of *k *markers, the union test is approximately *k*-1 times faster than the corresponding single marker test, because the computational burden of taking the union is less than the computational burden of performing one Fisher's exact test.

## Discussion

Recent attempts at elucidating the underlying genetic structure of common, complex diseases have been hampered by the use of methods that do not account for correlation in the data, are overly stringent, and are underpowered in the presence of allelic or locus heterogeneity and rare variants. Compared to other multi-locus methods designed to address these issues, unions offer many advantages, including accounting for linkage disequilibrium, epistatic interactions within (but not between) unions, and haplotype structure, with no requirement for the estimation of haplotypes or phase. Union testing can be viewed as a generalization of grouping schemes for analysis of independent rare variants [3,4] that allows for arbitrary patterns of correlation among common and/or rare variants. An additional advantage is that the counting algorithm implicitly accounts for correlation without explicit and potentially biased estimation of linkage disequilibrium measures and without the need of permutation testing to establish correct type I error rates. Union tests can be more powerful than single marker tests because they make fuller use of the information (*i.e*., genotype frequencies and multi-locus linkage disequilibrium [16]) in a multivariable framework. Power may also be gained by collapsing multi-locus genotype information into a single degree of freedom test [3].

The union test is a composite test of association of genotype frequencies and differential correlation among markers. Union tests are also haplotype tests and are sensitive to processes such as natural selection and population structure that may lead to differential correlation among markers. Previous analyses did not detect substantial population structure in either PD data set [7]. However, given that our multi-locus method is more powerful than single marker analysis under many genotypic configurations, it is possible that our method is more sensitive to cryptic relatedness or residual population stratification than is single marker analysis. To better address this issue, regression with a covariate for parental ancestry provides a framework compatible with unions [17].

Our proposed method is very flexible. The choice of which SNPs to combine into unions belongs to the investigator. We chose to combine consecutive SNPs in non-overlapping sets in order to take advantage of linkage disequilibrium, so that union testing may be viewed as a generalization of haplotype testing, while minimizing the correlation between tests. With an appropriate grouping scheme, unions may be used to address allelic heterogeneity, which occurs when the phenotype of interest is caused by different mutations within the same gene [18]. The role of allelic heterogeneity in complex disease is exemplified by the several, individually rare mutations in *NOD2 *associated with Crohn's disease [19]. Alternatively, one could focus on rare variants and group SNPs with minor allele frequencies < 5%. Rare variants have been predicted to play an important role in complex diseases [20], as has been illustrated for colorectal cancer [21] and cholesterol metabolism [22]. Unions may also be used to address genetic heterogeneity, which occurs when multiple genes cause the same disease.

Although we tested unions using contingency table analysis, it is straightforward to adapt our coding for use with generalized linear models and thus to incorporate covariates into the analysis. Our method is also flexible with respect to genetic models, such that coding for SNPs may be any combination of dominant, additive, or recessive [23]. Depending on the implementation (*e.g*., non-overlapping windows), union testing can also reduce the testing burden compared to single marker analysis. However, instead of non-overlapping windows as we used herein, a sliding window approach could be employed, at the cost of a higher testing burden.

There are two main disadvantages of unions. One disadvantage is that the SNP(s) and/or haplotype(s) within a union driving association cannot be inferred. If genome-wide analysis is intended as exploratory and hypothesis-generating, then this disadvantage is not a concern. The second disadvantage is that there are scenarios under which statistical power may be lost, depending on how collapsing is achieved. As with other collapsing methods, the power of union testing decreases if the size of the union is too large, whether or not the included variants are causal [3]. This limitation is more pronounced for markers with large minor allele frequencies. Similarly, the power of union testing decreases if both risk-increasing and risk-decreasing effects are included within a union. One approach to address both attenuation and cancellation of signal is to use a mixture model with one component for risk-increasing effects, one component for risk-decreasing effects, and one component for null effects.

We illustrated union testing by reanalyzing genome-wide genotype data for Parkinson disease. As expected, our findings represent a superset of those originally described [9]. SNP rs9310784 is 105 kb upstream of the gene *NGLY1 *(GeneID 55768), which encodes an enzyme thought to participate in proteasomal degradation of misfolded glycoproteins [24]. SNP rs1564282 is intronic in the gene *GAK *(GeneID 2580), which regulates clathrin-mediated membrane trafficking [25]. In the original analysis of these data, *GAK *was found to be associated under an additive model [9], although our analysis revealed that the association at this locus resulted from a dominant effect. Linkage disequilibrium levels are moderate to strong over a 186 kb region including *GAK *as well as five other genes, leading to poor resolution at this locus. SNPs rs1159220 and rs5998577 are intronic in the gene *SYN3 *(GeneID 8224). Synapsins are essential for assembly of synaptic vesicles and modulate neurotransmitter release, with expression of SYN3 being neuron-specific [26]. As with other genome-wide association studies, the power of our analysis was limited by small effect sizes and small sample sizes, particularly a small replication sample.

We have developed a unified framework using unions of multi-locus genotypes to powerfully, flexibly, and efficiently analyze genome-wide genotype data, candidate gene data, or medical sequencing data. Implementation with standard statistical packages (we used R) is straightforward. We replicated as significantly affecting PD risk a single, low-frequency haplotype in the region of the *GAK *gene on chromosome 4 and identified two other regions with suggestive evidence of multiple low-frequency risk haplotypes.

## Conclusions

Recent attempts at elucidating the genetic architecture of complex traits have been hampered by the use of methods that do not account for correlation in the data, are overly stringent, and are underpowered in the presence of allelic or locus heterogeneity and rare variants. To address these issues, we developed a powerful and efficient multivariable method based on unions of variants. Our method produces multivariable test statistics with reduced degrees of freedom compared to haplotype-based methods, implicitly accounts for linkage disequilibrium, and reduces the testing burden. Our method also generalizes collapsing methods as previously described for analysis of rare variants. Thus, we provide a unified framework for the analysis of one or more variants with any pattern of linkage disequilibrium and with any minor genotype frequency. Our method is suitable for genome-wide genotype data, candidate gene data, exome sequencing data, and whole genome sequencing data. Using our new method, we found evidence supporting susceptibility to PD at three loci.

## Authors' contributions

DS designed the study, analyzed the data, and drafted the manuscript. LKV conceived of the study and contributed to the interpretation of the results and to the writing of the manuscript. All authors read and approved the final manuscript.

## Supplementary Material

Additional File 1 **Table comparing the validity of the union test and the kernel machine-based test**. See the legend for Figure 1A for details. Click here for file

Additional File 2 **Table comparing the power of the union test and the kernel machine-based test for multiple independent markers with moderate effects**. See the legend for Figure 1B for details. Click here for file

Additional File 3 **Table comparing the power of the union test and the kernel machine-based test for multiple independent markers with larger effects**. See the legend for Figure 1C for details. Click here for file

Additional File 4 **Table comparing the power of the union test and the kernel machine-based test for markers with opposing effects**. See the legend for Figure 1D for details. Click here for file

Additional File 5 **Table comparing the power of the union test and the kernel machine-based test for dilute signal**. See the legend for Figure 1E for details. Click here for file

Additional File 6 **Table comparing the power of the union test and the kernel machine-based test for correlated predictors**.See the legend for Figure 1F for details. Click here for file

Additional File 7 **Table comparing the power of the union test and the kernel machine-based test for epistasis**. See the legend for Figure 1G for details. Click here for file

Additional File 8 **Table comparing the power of the union test and the kernel machine-based test for differential correlation between markers**. See the legend for Figure 1H for details. Click here for file

Additional File 9 **Quantile-quantile plots for genomic control**. A) The discovery sample. B) The replication sample. The red lines indicate the expected distribution. The inflation factors are shown, indicated by λ_GC_. Click here for file
