# Supplementary figures and images for: A unified framework for multi-locus association analysis of both common and rare variants
Source: BMC Genomics. 2011 Jan 31;12:89. doi: 10.1186/1471-2164-12-89 (PMC3040731; doi:10.1186/1471-2164-12-89)

**A**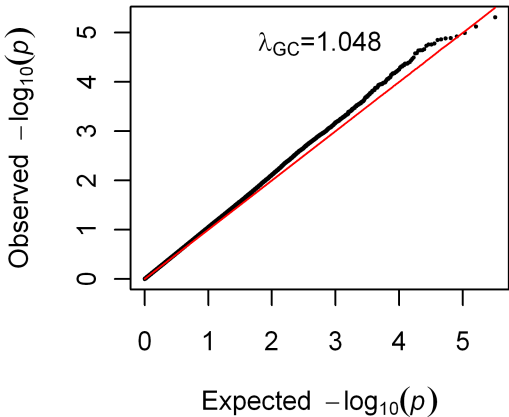**B**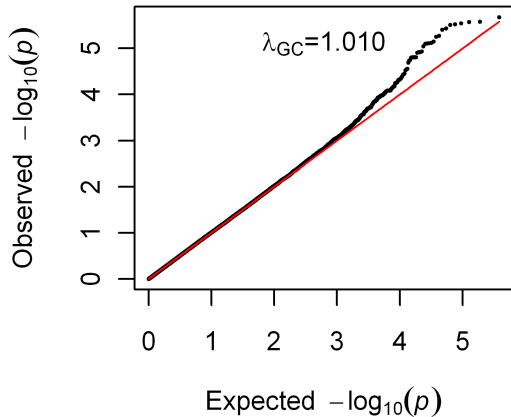

Supplement: Additional File 9 — Quantile-quantile plots for genomic control. A) The discovery sample. B) The replication sample. The red lines indicate the expected distribution. The inflation factors are shown, indicated by λGC. [file 1471-2164-12-89-S9.PDF]
